# Metabolic scaling of fire ants (*Solenopsis invicta*) engaged in collective behaviors

**DOI:** 10.1242/bio.059076

**Published:** 2022-02-28

**Authors:** Hungtang Ko, Keyana Komilian, James S. Waters, David L. Hu

**Affiliations:** 1 Woodruff School of Mechanical Engineering, Georgia Institute of Technology, 30332 Atlanta, GA, USA; 2 Coulter Department of Biomedical Engineering, Georgia Institute of Technology, 30332 Atlanta, GA, USA; 3 Department of Biology, Providence College, 02918 Providence, Rhode Island, USA; 4 School of Biological Sciences, Georgia Institute of Technology, 30332 Atlanta, GA, USA

**Keywords:** Metabolism, Scaling, Collective behaviors, Allometry

## Abstract

During flash floods, fire ants (*Solenopsis invicta* Buren) link their bodies together to build rafts to stay afloat, and towers to anchor onto floating vegetation. Can such challenging conditions facilitate synchronization and coordination, resulting in energy savings per capita? To understand how stress affects metabolic rate, we used constant-volume respirometry to measure the metabolism of fire ant workers. Group metabolic rates were measured in a series of conditions: at normal state, at three elevated temperatures, during rafting, and during tower-building. We hypothesized that the metabolic rate of ants at various temperatures would scale isometrically (proportionally with the group mass). Indeed, we found metabolic rates scaled isometrically under all temperature conditions, giving evidence that groups of ants differ from entire colonies, which scale allometrically. We then hypothesized that the metabolism of ants engaged in rafting and tower-building would scale allometrically. We found partial evidence for this hypothesis: ants rafting for short times had allometric metabolic rates, but this effect vanished after 30 min. Rafting for long times and tower-building both scaled isometrically. Tower-building consumed the same energy per capita as ants in their normal state. Rafting ants consumed almost 43% more energy than ants in their normal state, with smaller rafts consuming more energy per capita. Together, our results suggest that stressful conditions requiring coordination can influence metabolic demand.

This article has an associated First Person interview with the first author of the paper.

## Abstract (precis)

**Summary:** We measured the metabolism of fire ant rafts engaged in collective behaviors such as tower and raft building. We found that except for rafting at early stages, the metabolism scales isometrically with group size, indicating no group benefit in metabolism.

## INTRODUCTION

Collective behaviors drive the emergence of complexity across biology, from the aggregations of unicellular life to the flocks and swarms of dynamic animal groups (Camazine et al., 2001; Couzin and Krause, 2003). Even without any hierarchical control or regulation, animal groups can exhibit new properties that individuals cannot achieve. Colonies of eusocial insects can exhibit emergent patterns in their foraging, immunity, division of labor, caste development, nest architecture, and decision making. Thus, eusocial insects have long been used as models for understanding the emergence of collective behaviors (Pacala et al., 1996; Gordon and Mehdiabadi, 1999; Jeanson et al., 2007; Holbrook et al., 2011; Morand-Ferron and Quinn, 2011; Dornhaus et al., 2012; Berdahl et al., 2013; Ulrich et al., 2018). While the energetics of individual ants during walking, running, and load-carrying have been studied (Lighton et al., 1987, 1993), little is known about the energetics of collective behaviors in animal groups. Increases in overall group size typically correspond with improved ability to maintain homeostasis and positive fitness. Do specific collective behaviors confer energetic savings to the colonies? Or have collective behaviors evolved despite having imposed relatively greater metabolic costs?

While the metabolic rates associated with collective behaviors have not been measured systematically, there is a growing literature on the scaling of metabolic rate with colony size in groups of social insects. The metabolic rates 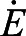 of animal collectives are usually characterized as power laws in terms of total body mass *M*, such as in:
(1)

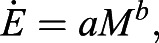
where the scaling coefficient *a* is a constant and the scaling exponent *b* ranges from 2/3 to 1 (Kleiber, 1932; Gillooly et al., 2001; Dodds et al., 2001; Brown et al., 2004; Glazier, 2010, 2014a,b, 2020). A number of studies have demonstrated that, much like the corresponding and widespread scaling pattern for unitary organisms, colony-wide metabolic rates exhibit allometric scaling or negative allometry (*b*<1). The mass-specific or per-capita metabolic rate decreases with colony size in ants, bees, termites, and others, suggesting a ‘group effect’ (Gallé, 1978; Hughes and Hughes, 1986; Nakaya et al., 2003; 2005; Hou et al., 2010; Gillooly et al., 2010; Fewell and Harrison, 2016; Waters et al., 2017; Burgess et al., 2017). While various theories over the last two decades have been raised to explain the ‘group effect’ (Glazier, 2005, 2010, 2014a,b, 2020), sufficient experimental evidence is often lacking. Thus, one of the motivations of this study is to provide an insect collective with a challenging task to elicit cooperation and group effects in metabolic rates.

When insect workers are isolated from their other colony members, the group effect vanishes (Brian, 1973; Lighton and Bartholomew, 1988; Lighton, 1989; Waters et al., 2010). For most of these measurements, the insects were not challenged with collective tasks. A notable exception is the overwintering honeybee colonies that swarm together to maintain a suitable core temperature for brood development (Heinrich, 1981; Southwick, 1985). This thermoregulatory collective behavior is likely to be influenced by surface area to volume ratio (Heinrich, 1981). Indeed, overwintering honeybee colonies exhibit striking metabolic allometry (Southwick, 1985) and spend less energy per capita in larger swarms.

Fire ants (*Solenopsis invicta*) are an excellent model system to study the metabolic costs associated with collective behaviors. Originally from the Pantanal region of Brazil and now spread across the tropics, fire ants display a range of collective behaviors (Tschinkel, 2013). To survive floods that are frequent in their natural habitat, they link their bodies to build waterproof rafts (Mlot et al., 2011). Haight (2006) showed that ants in rafts increase the amount of venom they inject, likely to compensate for their highly vulnerable state. This defensive state suggests a higher activity level and potentially higher metabolic rate. At the same time, fire ant rafts have been documented to float into larger waters and even the open ocean (Adams et al., 2011). The potential for long-term starvation of rafting ants indicates that a lower metabolic rate could also be advantageous. The tradeoffs for increasing or decreasing metabolic rate naturally lead to the question addressed here: what is the metabolic rate of rafting ants?

When the ant raft encounters vegetation such as tall grasses, it morphs into an ant tower, which serves both as an anchor and a temporary encampment until the water subsides (Phonekeo et al., 2017). Such towers can reach heights of 10 cm and are characteristically narrow at the top and bell-shaped at the bottom in order to more equally distribute the weights of ants in the tower. When ants in captivity are deprived of underground nesting sites, they build towers on walls or vertical protrusions such as test tubes, sticks, and nails. X-ray videography has shown that these towers are perpetually dismantled and reconstructed (Phonekeo et al., 2017). Compared to dispersed groups of fire ants, both ant rafts and towers require higher levels of cooperation. It is plausible that performing these collective tasks reduces the relative demand from individuals, hence inducing metabolic allometry.

Colonies of fire ants can be collected in the field, reared in the laboratory, and experimentally induced to perform rafting and tower-building behaviors on demand. In this paper, we present our measurements of metabolic rates in groups of fire ants engaged in collective behavior and compare these results with those from groups at rest and across a range of temperatures. We hypothesize that when ant workers are resting, their metabolic rates are primarily associated with baseline maintenance costs and are additive; the metabolic scaling should thus be isometric. However, when they are engaged in collective tasks such as building and maintaining rafts or towers, they should exhibit group effects and thus negative allometry. By considering the scaling of metabolic rate in these contexts, we aim to identify metabolic advantages associated with work organization in this species.

## RESULTS

### Fire ants in elevated temperature obey isometric metabolic scaling

**Fig. 1.**
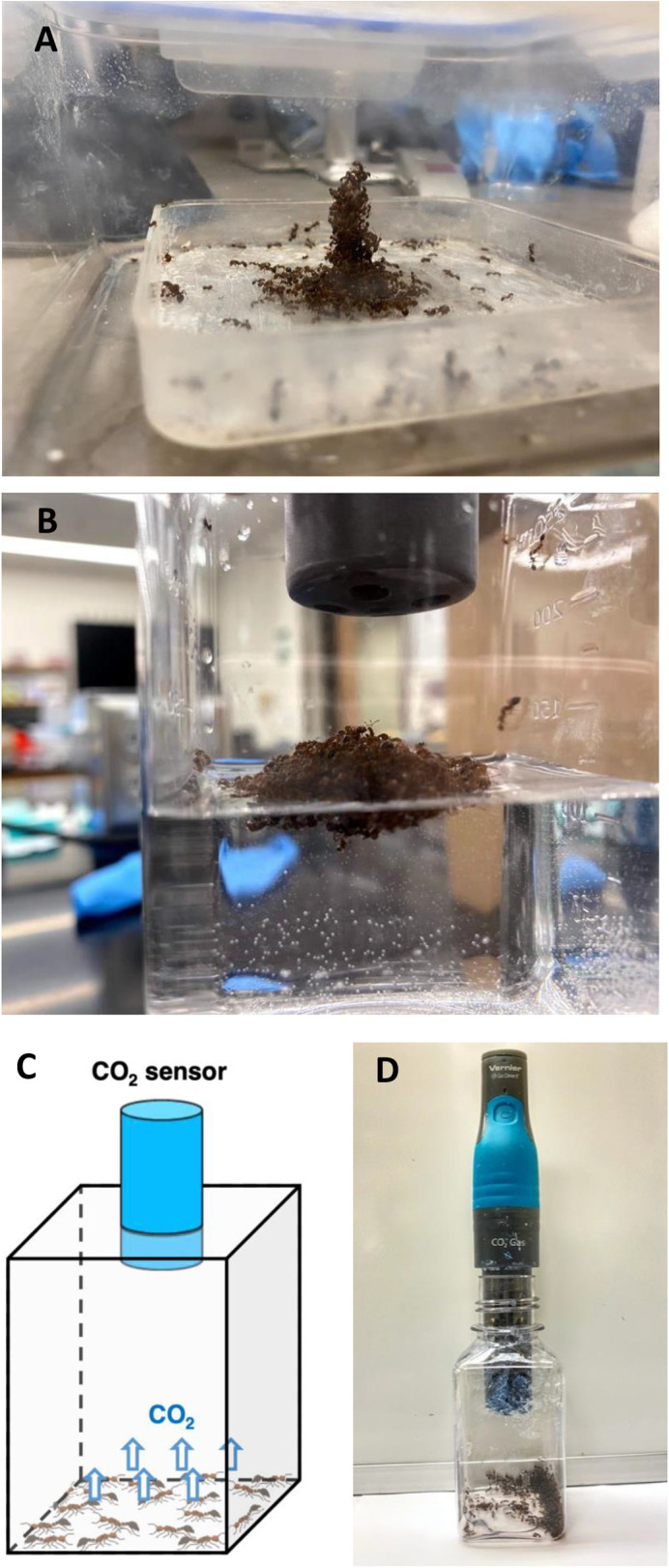
**Constant-volume respirometry measurement of fire ants.** (A) An ant tower composed of 630 ants surrounding a hydrophobic central rod. (B) A raft composed of 1500 ants during respirometry measurement. A schematic (C) and (D) photo of the experimental setup.

**Fig. 2.**
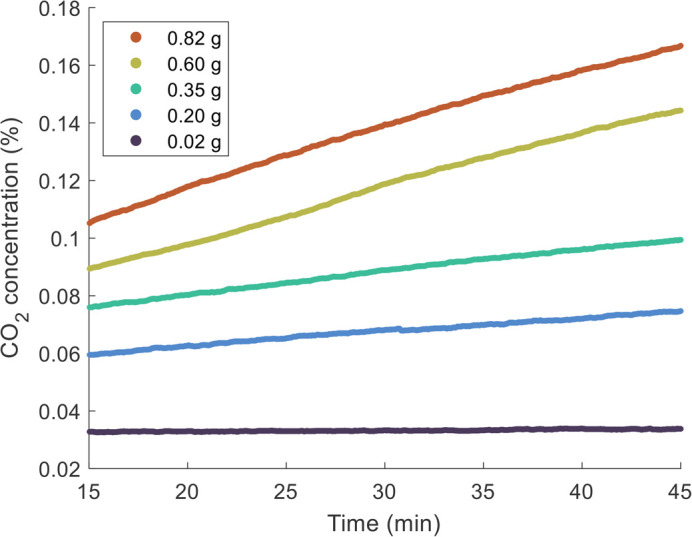
**The time course of CO_2_ for five representative trials.** CO_2_ concentration in the closed chamber increases linearly with time. The time-rate of change of CO_2_ concentration increases with the number of ants. Ants in this data set were at room temperature (24°C) on dry land.

We performed 68 trials of carbon dioxide measurement on groups of fire ant workers ranging from 20 to 2000 ants (0.02 g to 2 g) (Fig. 1). We begin with the time course of raw data collected by our CO_2_ sensor. Fig. 2 shows the steady increase of CO_2_ concentration for five masses of ants (0.02 g to 0.82 g) at room temperature (24°C), from t=15 min to 45 min. As detailed in the methods section, we estimate that it takes around one minute for CO_2_ to diffuse from the ants to the sensor. To ensure the chamber is adequately mixed, from hereon we report data 15 min after insertion of the sensor. The constant slope for all samples indicates steady-state behavior. Even for the smallest number of ants studied, 20 ants equivalent to 0.02 g, the slope of CO_2_ concentration increase is significant at 3.64×10^−5^ %/s(*R*^2^=0.86, *P*<10^−5^). From hereon, we convert carbon dioxide production rate to metabolic rate 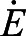 using Eqn 2 in the Materials and Methods.

**Fig. 3.**
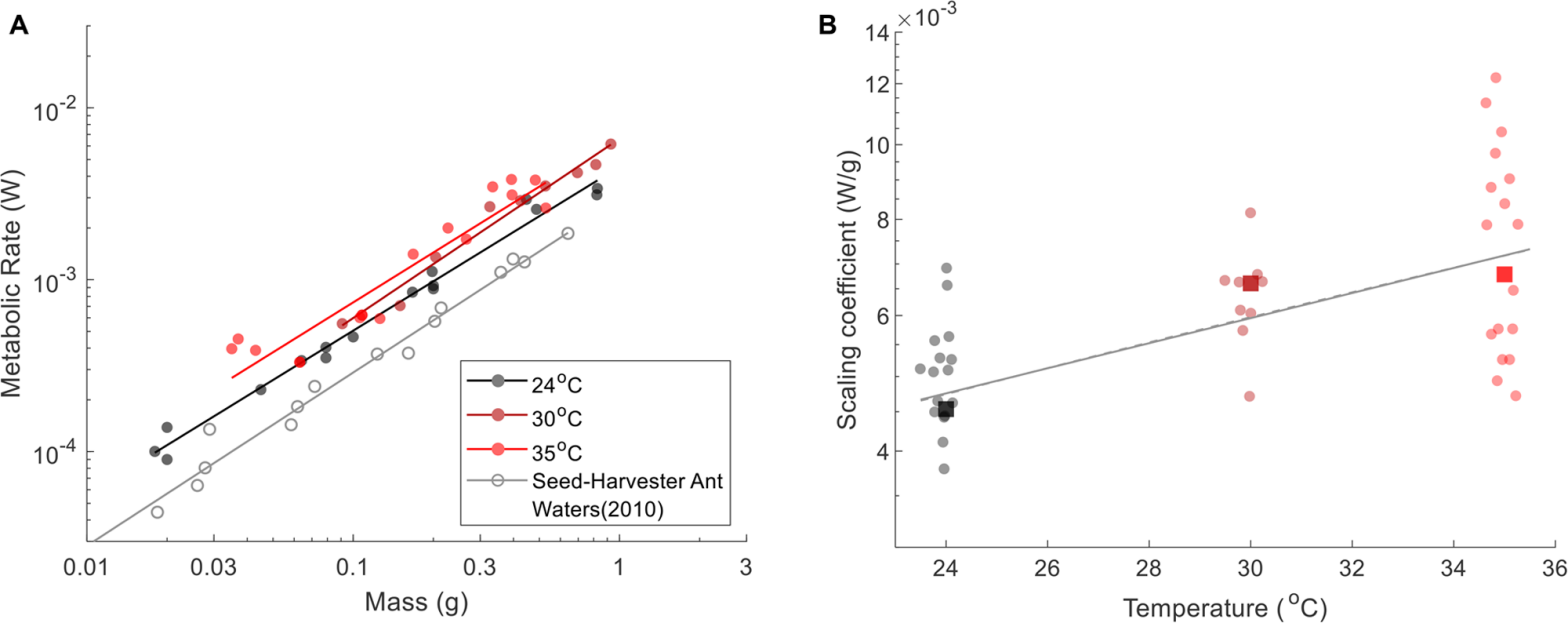
**Effects of temperature on fire ant metabolic rate.** (A) Metabolic rate of ant workers 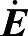 scales linearly with mass of ant worker groups ***M*** at 24°C, 30°C, and 35°C on dry land. The scaling exponents range from 0.95 to 1.04 (see Table 1). Our measurements, the solid circles, compare well with previously reported data with workers groups of seed-harvest ants (open circles) (Waters et al., 2010). (B) Scaling coefficient ***a*** (squares) increases with temperature. The data are fit to Eqn 3 (solid line) and Eqn 4 (dash line), resulting in *Q*_10_=1.45 and *E_A_*=0.30 eV, respectively. Due to isometric scaling, the mass-specific metabolic rates 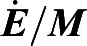 (shown by the solid circles) are close to ***a***. Artificial jitters are added in the horizontal direction for better data visualization.

Next, we consider mild stress due to small temperature increases. These trends will be an important baseline to compare our rafting and towering treatments. Fig. 3A shows the mass dependence of metabolic rates for three different temperatures (24°C, 30°C, and 35°C). For all the temperatures observed, scaling exponents *b* were close to 1, indicating that the metabolic scaling is isometric (Table 1). Indeed, statistical tests showed that for our room temperature (24°C) metabolic rates, the exponent *b* is significantly larger than 0.75 (*p*=1.4×10^−5^) and indistinguishable from 1 (*p*=0.16).

**Table 1.**
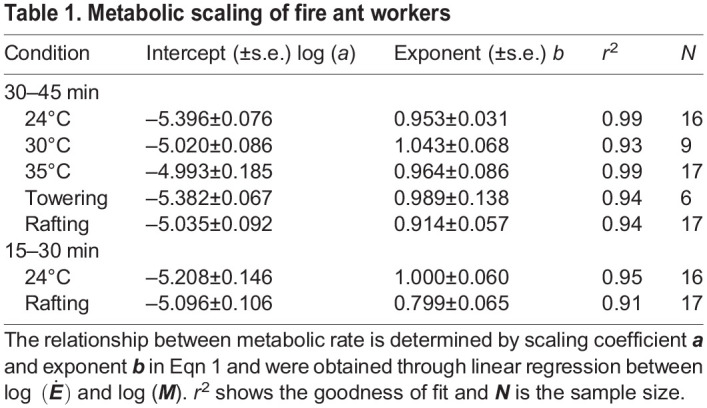
Metabolic scaling of fire ant workers

Fig. 3A also demonstrates that metabolic rate increases with temperature. This effect can be quantified by comparing the y-intercepts for each trend line, which are equivalent to the natural log of scaling coefficient *a* in Eqn 1. Higher temperatures are associated with higher scaling coefficients *a*. Fig. 3B shows that scaling coefficient *a* increases with temperature. By using Eqn 3 and Eqn 4 in the Materials and Methods, one can obtain the temperature coefficient *Q*_10_=1.45 and activation energy *E*_*A*_=0.30 eV. The fit lines for the temperature coefficient *Q*_10_ and activation energy *E*_*A*_ models are shown by the solid and dashed lines, respectively, and are virtually identical. Since metabolic rate scales isometrically with mass, scaling coefficient *a* is equivalent to the mass-specific metabolic rate 
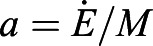
. Indeed, in Fig. 3B, mass-specific metabolic rates (dots) of individual trials roughly match the scaling coefficients *a* (squares).

### Fire ants building rafts and towers show some evidence of negative allometric metabolic scaling

**Fig. 4.**
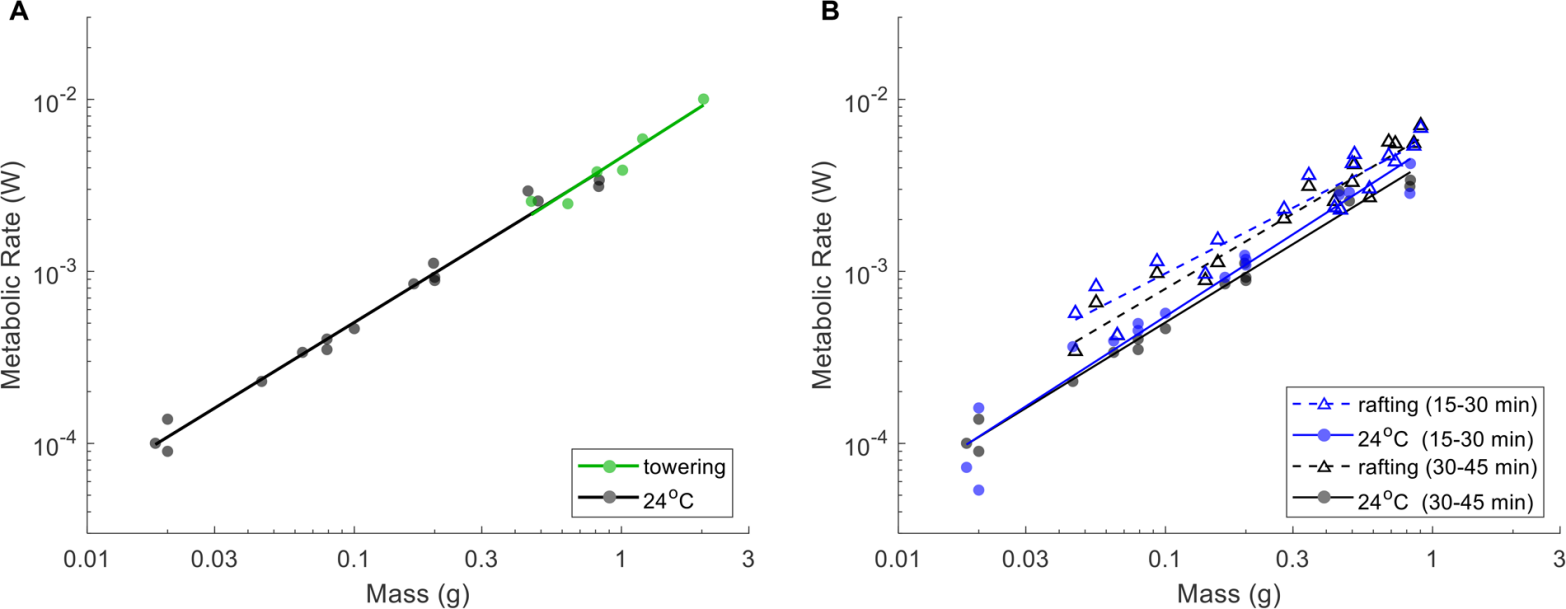
**Metabolic rats of ant towers and ant rafts.** (A) Time course of the metabolic rate of towers (green points) and free-roaming ants (gray points). (B) Time course of metabolic rate for ant rafts at two different time frames. Early times (15–30 min) are given by the blue triangles. Late times (30–45 min) are given by the grey triangles. Similar time frames are given for free-roaming ants but with blue and gray circles, respectively.

Our experiments with rafts and towers partially supported the hypothesis that ants performing cooperative behaviors exhibit a group effect. We first discuss ant towers and then proceed to ant rafts. We observed six successfully built towers in our custom-made metabolic chamber. Fig. 4A shows the metabolic rate 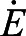 for fire ant towers and compares them to the room temperature data from Fig. 3A. Not only is the tower scaling isometric, but the scaling trend is also almost indistinguishable from free-roaming ants at room temperature (24°C). Note that our experiments did not measure the energetic cost to build the tower because our protocol requires us to wait 15 min after ants were in the metabolic chamber, at which time the tower was already constructed. Thus our data only reflects the energetic costs to maintain a tower long after it had been built. Previous work with x-ray videography showed that ant towers sink at a rate of 0.38 mm/min (Phonekeo et al., 2017). Therefore, our measurement shows the metabolic energy required for ants to maintain and rebuild the tower is negligible compared to normal activity. Phonekeo et al. (2017) also suggested that towers were simply an example of three-dimensional exploration. As such, our results here suggest that three-dimensional and two-dimensional exploration require the same energy expenditure.

Rafting ants, on the other hand, consume considerably more energy. Fig. 4B and Table 1 show that rafting ants during the 30–45 min observation period have a 43% higher metabolic rate than ants on dry land. During this period, the metabolic rates of rafts scale isometrically. The increased metabolism of rafting ants is consistent with our observation of many of the rafting ants flailing their legs. The 43% larger scaling coefficient *a* for rafts is equivalent to the ants subjected to a temperature of 32.3°C, according to Eqn 3 and Fig. 3B.

Fig. 4B shows the time dependence of the ant rafts' metabolic rate. We characterize this time dependence by comparing the scaling trend lines for the 15–30 min (blue dashed line) and 30–45 min time intervals (black dashed line). Note that these trend lines are each characterized by *N*=17 experiments and are an excellent fit to the data (*R*^2^=0.91−0.94). We first consider small rafts. According to the trend lines, the smallest raft in our data (64 ants or 0.046 g) spent 66% less energy in the 30–45 min window than in the 15–30 min window. Thus, small rafts have metabolic rates that improve with time spent on the water surface: this might be associated with the ants being in a frantic state at first and then settling down. However, as the raft increases in size, the time dependence is reduced. A large raft of 530 ants (0.53 g) no longer shows time dependence: the 15–30 min interval and 30–45 min intervals both yield the same metabolic rates. This feature is shown by the intersection of the trendlines. Due to the change in time dependence with raft size, the scaling exponent *b* increases from 0.80 to 0.91, shifting from negative allometry (15–30 min) to isometry (30–45 min). As the ants settle down, they continue to maintain a 43% higher metabolism than ants on land, and the energy use per capita becomes independent of raft size.

## DISCUSSION

Our hypothesis was that fire ants maintaining rafts and towers should exhibit allometric scaling due to their cooperation during these tasks. This hypothesis was only partially supported. Energy consumption per capita remains the same for most of the test cases studied, except for when ants just started to make rafts. Thus, our study highlights the challenges faced by small ant rafts, at least in the initial stages of raft construction. In this phase, ant rafts exhibit metabolic scaling exponents of 0.8, which indicates that small rafts use higher energy per capita than larger ant rafts. This result is biologically relevant because large rafts may break into small rafts as they encounter obstacles or turbulent waters. Future researchers might try to link the observed group effect to particular behaviors. Perhaps smaller rafts have more trouble making stable structures due to their lessened capillary effects. Specifically, the attraction between floating objects on the water surface scales with the net weight of the objects, a phenomenon known as the ‘Cheerios effect’ (Vella and Mahadevan, 2005). Smaller rafts have less capillary attraction to keep the raft together and may require the ants to swim more actively to prevent separation.

Future work should aim to determine a mechanism for the allometric metabolic rates for small ant rafts. Insect literature (Waters and Harrison, 2012; Chown et al., 2007) classifies metabolic rates into routine, standard, field, active, inactive, and resting. When measuring individuals, rigor can be added to classifies their respiratory pattern (e.g. cyclic or discontinuous gas exchange) and activity levels (e.g. walking velocities), but this is more difficult for large groups. To correlate activity levels and metabolic rates for insect collectives, future workers would need to perform simultaneous metabolic measurement and videography, which was not possible with our current setup.

Our study showed that the maintenance of towers costs less energy than rafts. This makes sense because towers are formed on dry land and on stable surfaces, whereas rafts are formed on unstable fluid surfaces. Maintaining a raft also has much higher stakes than a tower. If part of the tower breaks and falls off, it can be rebuilt. In fact, tower failures are often observed throughout construction. However, if an ant raft breaks apart, the pieces are subject to surrounding currents, and the raft may drift apart, greatly diminishing the chances of the raft rejoining again. Raft maintenance is thus much more demanding and life-threatening, which is consistent with its higher associated metabolism.

When we make comparisons to studies of fire ants and seed harvester ants, we find that our results are consistent. Waters et al. (2010) studied groups of seed harvester ant workers. They found isometric scaling for group sizes manipulated in the laboratory, but not for colonies of various unmanipulated sizes. By comparing scaling coefficients *a*, we find that at room temperature, fire ants' metabolism is about 1.5 times higher than seed harvester ants. Moreover, our temperature coefficient *Q*_10_=1.45value for fire ant groups is comparable to previous *Q*_10_ measurements for individual fire ants, which are 1.6 at 25°C and 1.3 at 30°C (Vogt and Appel, 2000). We thus conclude that our low-cost closed volume respirometry equipment provided results consistent with other groups. Our experimental setup would be suitable for use in undergraduate classes.

### Conclusion

In this study, we performed experiments to measure the scaling of metabolic rates of groups of fire ant workers. We measured the carbon dioxide production rate associated with various temperatures and dry and wet conditions. In nearly all cases, we found that metabolic rate scales isometrically, or proportionally with the number of ants, indicating that fire ant workers spend the same energy per capita across group sizes. The one instance that we found for a group effect, as shown by allometric metabolic rates, was the initial construction phase of fire ant rafts. Small ant rafts expend more energy than large ant rafts, possibly due to the greater difficulty in keeping small rafts together.

## MATERIALS AND METHODS

### Fire ant collection and maintenance

We collected red imported fire ants *Solenopsis invicta* from the campus of Georgia Institute of Technology, Atlanta, USA, throughout the year in 2020. We dug up ant mounds and isolated ants from the soil by simulating rainfall using drip floating methods (Banks et al., 1981). Both queens and brood were present in all our lab colonies and experiments were conducted within 3 months after ants were removed from the wild. Ant colonies lived in a large plastic container with enclosed Petri dishes as artificial nests. Ants were fed with excess water and honey jelly, intermittently supplemented with black soldier fly larvae, and dried crickets as a protein source. We randomly picked ant workers from the colony and immediately commenced experiments.

In nature, ant rafts and towers often include brood, which the ants rescue from underground chambers when evacuating. These brood have a high fat content, so they are often placed on the bottom of the raft to help keep it afloat (Adams et al., 2011). Despite their utility, we did not include brood in our experiments because of the uncertainty associated with brood. Due to the emergency nature of the evacuation, the number of brood included in the raft can vary. Moreover, the age of the brood (egg, larva, pupae) may influence their pheromone levels and our resulting measurements. Lastly, as shown in previous work, fire ants easily build towers and rafts without brood (Mlot et al., 2011; Phonekeo et al., 2017; Adams et al., 2011). Thus, for uniformity, all experiments in this study are performed with only worker ants and excluding brood.

### Metabolic rate measurement at elevated temperatures

We used constant volume respirometry to measure fire ant metabolism (Fig. 1). The carbon dioxide concentration was monitored using the Vernier Go Direct CO_2_ sensor. We procured 0.2% and 1% carbon dioxide mixtures from GASCO and calibrated the sensors using a two-point calibration method. Measurements began when we transferred the ant workers to the Vernier 250 ml chamber and inserted the gas sensor to create an airtight seal. Recording started immediately afterward and continued for 45 min, with data taken every 2 s. The first 15 min of data were discarded as the ants settled into their new surroundings. All our raw measurements are under 0.5% CO_2_, which is well below the concentration range reported to affect insect behaviors (Nicolas and Sillans, 1989). Thus we conclude that our carbon dioxide measurements are not affected by the accumulation of carbon dioxide in the container.

At higher temperatures, ants are stressed, increasing their metabolic rates. How does temperature affect the metabolic scaling with mass? We conducted experiments at 24°C, 30°C, and 35°C using a hot water bath, which was heated from an underlying hot plate to the desired temperature. The Vernier 250 ml chamber was submerged entirely in the bath and hot-glued to the bottom of the bath to prevent it from floating. We confirmed the temperature ants experienced by cross-checking across four independent measurements, including a hot plate temperature sensor, Mercury-in-glass thermometer in the water bath, the temperature reading from the Vernier Go Direct CO_2_ sensor, and a Physitemp IT-18 Flexible Implantable Microprobe at the bottom of the chamber, right next to the ants. At equilibrium, when we conducted our measurements, all four readings were within 0.5°C of each other, which confirms that we were generating a consistent and accurate temperature.

### Metabolic data processing and statistics

The rate of CO_2_ accumulation was calculated by fitting a linear regression to the time series data on CO_2_ concentration for two separate periods: 15–30 min and 30–45 min after the ants had been transferred into the container. The fitting was done directly in the Vernier graphical analysis software.

We convert the rate of CO_2_ concentration increase 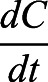 to metabolic rate 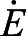 using:
(2)

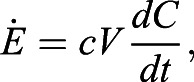
Here *c*=28 J/ml is the energy equivalent of carbon dioxide, which was found using flow-through respirometry of desert ants *Cataglyphis bicolor* (Lighton and Wehner, 1993). The volume of the container *V*=*AH* is the product of the base area *A* and the height *H*. In this conversion, we assume that fire ant's respiratory exchange ratio (RER), defined as the ratio of production of carbon dioxide to the uptake of oxygen, is 0.7 (Lighton et al., 1987; Lighton and Wehner, 1993), which indicates the ants are using both carbohydrate and fat fuel sources. It will take future workers to measure simultaneous oxygen and carbon dioxide for fire ants and validate this RER and the *c* value for fire ants engaged in collective behaviors.

Finally, we performed a linear regression between the logarithm of metabolic rate 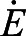 and the logarithm of mass *M* and obtained the prefactor *a* and the exponent *b* in MATLAB. Here we have assumed a constant respiratory quotient of 0.71 and therefore a constant energy equivalent *c* (Lighton and Wehner, 1993).

The two data sets' slopes (taken from the two time periods mentioned previously) did not show a significant difference in all cases except for rafting ants (see Table S1). To further confirm that the metabolic rate is not impacted by the time period of choice, we performed ANCOVA analysis in MATLAB. We found that the influence of the fitting period on the mass scaling is insignificant. The *p*-value, which suggests the possibility that the effect is trivial, ranges from 0.46 to 0.62 for conditions other than rafting. As demonstrated in the result sections we did identify a weak time dependence of metabolic scaling of rafts, with a *p*-value lower at 0.19. In this paper, unless otherwise noted, data from the 30–45 min time frame was used.

### Rafting and towering

Experiments were conducted at room temperature (24°C). For both rafting and towering experiments, we used previously established protocols (Mlot et al., 2011; Phonekeo et al., 2017) for generating these aggregations in the lab, which we review briefly below.

For the rafting experiments, we first transferred 75 ml water at room temperature to the 250 ml chamber, which provided a 57 mm×57 mm square-shaped water surface for the ants to raft on. Before each experiment, we collected ants in a beaker and swirled them for the ants to aggregate into a dense ball. After we dumped it onto the water surface, the ball of ants expanded into the ant raft (Fig. 1B). We performed 25 rafting experiments, and 17 were successful. A successful trial was one in which the ants formed a raft within the first 3 min of the ball being placed on the water surface. Failed trials involved ants spreading out and not aggregating into a raft (i.e. with individual ants each swimming freely). Metabolic measurements of failed trials were not recorded.

To conduct ant tower experiments, we customized a larger, taller chamber to house the tower (Fig. 1A). The base of the container consisted of a 1030 ml airtight glass food container with a plastic lid. We cut the lid and attached it to the top of the plastic bottle that accompanied the sensor. We tested the airtight seal by submerging the setup into a water bath.

To create towers, we placed ants in a beaker and shook them into a dense ball, which was then transferred into the custom chamber. Immediately after adding the ants, a 25 mm long 2.5 mm in diameter nail coated in Fluon was inserted into the middle of the ball to encourage the ants to surround and build a tower around it. When the tower was built, the lid of the container was closed, the sensor inserted, and the data collection began.

We briefly justify the use of the nail as the central rod here. Without a solid object to build on, ants will not build a tower. The nail's flat end, of diameter 10 mm, allowed it to stand on its own on the bottom of the container while the ants towered around it. Our previous study (Phonekeo et al., 2017) used a talc powder-coated central rod, but the Fluon coated nail serves the same purpose. Both make it difficult for ants to climb. While the properties of the nail may deviate from natural vegetation, we use a hydrophobic rod to start with a blank slate to ensure repeatable experiments. To build a tower on the hydrophobic rod, ants compensate for lack of grip by connecting their bodies to form a stack of rings around the rod to build the tower.

We attempted 12 towering trials, of which six were successful. A successful experiment is one in which ant towers formed quickly and of sufficient height. Specifically, the tower had to be fully built within 8–10 min of the nail insertion, and the tower's height had to reach the entire height of the nail, 25 mm. Since the length of a single ant is 3 mm, a tower of 25 mm is about eight ants tall when ants are oriented vertically. Towers were wider at the base and narrow at the top. The individual ants were arranged roughly vertically around the tower, with ants walking both up and down the sides. All towers that formed in this manner maintained their shape for the entire duration of the 45-min experiment. Failed trials were distinct from towering behavior: ants would not show any interest in the nail. Instead, they spread out evenly in the container. Under those circumstances, the ants were returned to the colony, and we restarted the tower-building with fresh ants from the same colony.

### Temperature dependence of metabolism

While it is widely acknowledged that metabolic rate increases with temperature, there are two methods to represent this effect in the literature: (1) temperature coefficient *Q*_10_ and (2) activation energy *E*_*A*_. Both variables are associated with a scaling coefficient *a* in Eqn 1, but each has different dependence in temperature. To obtain a constant *Q*_10_, a linear relationship between ln *a* and temperature *T* is assumed; for *E*_*A*_, a linear relationship between ln *a* and 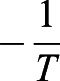 is assumed:
(3)

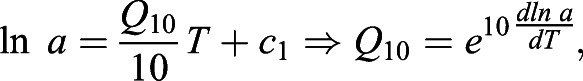

(4)

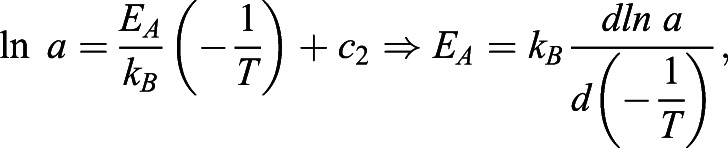
where *k*_*B*_ is the Boltzmann's constant (8.62×10^−5^ eV/K) and *c*_1_, *c*_2_ are fitting constants. *Q*_10_ and *E*_*A*_ only concern the slope of linear relationship. Therefore, y-intercepts *c*_1_ and *c*_2_ are irrelevant in the calculation.

Despite the wide use, many researchers found *Q*_10_ to be temperature-dependent itself. In fact, Eqn 3 predicts *Q*_10_ to decrease with temperature, which is consistent with the measurements in Lighton and Bartholomew (1988); Nielsen et al. (1999); Shik et al. (2019). Our data at the three temperatures were insufficient to favor either model. Therefore, we report both temperature coefficient *Q*_10_ and activation energy *E*_*A*_ using Eqns 3 and 4.

### Diffusion time for constant volume respirometry

After the ants were transferred to the container and the CO_2_ sensor inserted, there was a transient period when the sensor reading was unstable. Three processes took place during this period: the ants explored the container, the sensor settled to the new air environment, and the newly produced CO_2_ was transported to the sensor. The last effect can be analyzed rigorously through our derivation below. Here, we show that the CO_2_ concentration increases at the same rate everywhere in the container after one minute, which is the diffusion time required by our constant volume respirometry setup.

We begin with the diffusion equation,
(5)

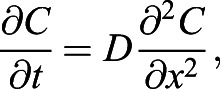
which determines the dynamics of *C*(*x*, *t*), the CO_2_ concentration field that varies with the vertical distance *x* from the sensor and time *t*. *D* is the diffusivity of CO_2_ in air. Diffusion equation, Eqn 5, is solved together with the boundary conditions. At the ants’ position, *x*=*H*, the CO_2_ production rate of the ants results in the CO_2_ influx 
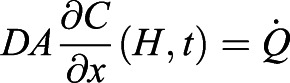
. Here *H* is the height and *A* is the cross-sectional area of the chamber, and 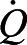 is the CO_2_ production rate which is proportional to the energy consumption rate 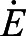. At the sensor position, *x*=0, gas cannot leave the container 
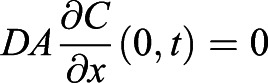
. The concentration field is initially a constant value *C*(*x*, 0)=*C*_0_. In order to solve for the diffusion equation, we introduce an auxiliary variable 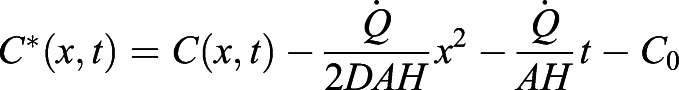. The new variable C*(*x*, *t*) has homogeneous boundary condition and can be solved analytically using the standard separation of variable technique. As a result, the solution can be written as:
(6)

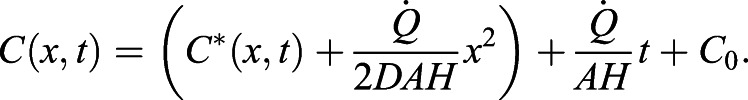
The auxiliary field C*(*x*, *t*) starts with 
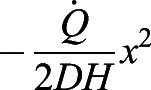
 at *t*=0, so that the value within the bracket in Eqn 6 starts at 0, and *C*(*x*, *t*) can start from *C*_0_. Then, C*(*x*, *t*) decays exponentially to a constant with the decaying time scale 
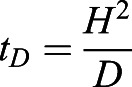
. In accordance with intuition, The diffusion time *t*_*D*_ increases with the distance between the ants and the sensor and decreases with diffusivity of gas. Our setup has height *H*=6.5 cm and the diffusivity of CO_2_, *D*=0.14 cm^2^/s. Therefore, *t*_*D*_ is around 5 min. After around *t*=*t*_*D*_, the whole concentration field will increase at the same rate 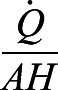, i.e. metabolic rate divided by the volume of the container.

Both analytical and numerical solutions to the concentration field *C*(*x*, *t*) can be obtained. We graphed the evolution of *C*(*x*, *t*) and C*(*x*, *t*) (Fig. S1) which shows that the initial transient effect dies off faster than *t*_*D*_. Based on our analytical solution and simulation, the transport of CO_2_ only took 0.2*t*_*D*_, or 1 min in our setup.

## Supplementary Material

Supplementary information
