# Supplementary material for: Metabolic scaling of fire ants (Solenopsis invicta) engaged in collective behaviors
Source: Biol Open. 2022 Feb 28;11(2):bio059076. doi: 10.1242/bio.059076 (PMC8905630; doi:10.1242/bio.059076)
Supplement: Supplementary information [file biolopen-11-059076-s1.pdf]

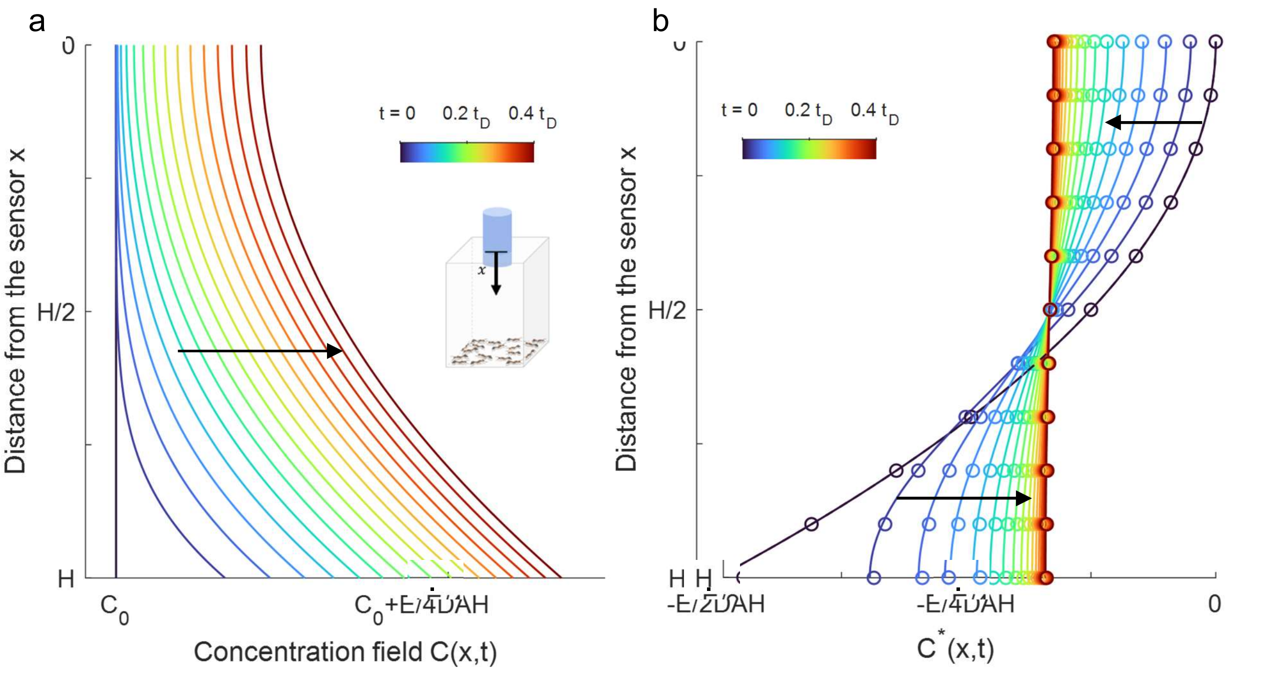

Fig. S1. (a) Evolution of the CO_2 concentration field C(x,t). After the initial buildup, the field increases at the same rate everywhere. (b) The auxiliary variable $C^*(x,t)$, which highlights the initial transient effect, converge to a constant value quickly. The time scale of the transient effect $t_D = H^2/D$. The hollow spots are numerical solutions while the solid lines are analytical solutions. The analytical solution shown only used the first 100 terms for summation. Dash line shows the initial condition $C^*(x,0) = -\frac{\dot{E}}{2DA}x^2$, which show excellent matching with the analytical solution.

Biology Open • Supplementary information

Table S1. Metabolic scaling of fire ant workers in various conditions

Condition	Fitting period	Intercept (± SE) $\log(a)$	Exponent (± SE) b	r^2	N
24°C	15-30 min	-5.208 ± 0.146	1.000 ± 0.060	0.95	16
24°C	30-45 min	-5.396 ± 0.076	0.953 ± 0.031	0.99	16
30°C	15-30 min	-4.821 ± 0.085	1.092 ± 0.067	0.89	9
30°C	30-45 min	-5.0198 ± 0.086	1.043 ± 0.068	0.93	9
35°C	15-30 min	-5.064 ± 0.249	0.851 ± 0.116	0.99	17
35°C	30-45 min	-4.993 ± 0.185	0.964 ± 0.086	0.99	17
Towering	15-30 min	-5.248 ± 0.049	1.114 ± 0.083	0.91	6
Towering	30-45 min	-5.382 ± 0.067	0.989 ± 0.138	0.94	6
Rafting	15-30 min	-5.096 ± 0.106	0.799 ± 0.065	0.91	17
Rafting	30-45 min	-5.035 ± 0.092	0.914 ± 0.057	0.94	17

Table S2. Raw data of metabolic rate in all trials

Condition	Mass(g)	Metabolic rate (W)	
		15 - 30 min	30-45 min
24ºC	0.079	4.53E-04	3.51E-04
	0.079	4.98E-04	4.04E-04
	0.198	1.24E-03	1.12E-03
	0.200	1.09E-03	8.85E-04
	0.822	2.84E-03	3.12E-03
	0.826	4.24E-03	3.39E-03

Biology Open • Supplementary information

	0.020	5.35E-05	8.99E-05
	0.045	3.65E-04	2.29E-04
	0.064	3.95E-04	3.38E-04
	0.100	5.71E-04	4.65E-04
	0.488	2.88E-03	2.57E-03
	0.447	2.79E-03	2.94E-03
	0.018	7.26E-05	1.00E-04
	0.020	1.61E-04	1.38E-04
	0.167	9.24E-04	8.47E-04
	0.200	1.17E-03	9.24E-04
	0.204	1.74E-03	1.35E-03
	0.326	2.48E-03	2.66E-03
	0.425	3.65E-03	2.88E-03
	0.527	4.09E-03	3.51E-03
30ºC	0.695	6.12E-03	4.20E-03
	0.091	5.05E-04	5.55E-04
	0.150	9.41E-04	7.07E-04
	0.815	5.19E-03	4.68E-03
	0.928	6.76E-03	6.15E-03
	0.266	1.60E-03	1.72E-03
	0.395	3.87E-03	3.11E-03
	0.168	8.69E-04	1.41E-03
	0.227	2.03E-03	2.00E-03
	0.334	3.73E-03	3.47E-03
35ºC	0.393	4.94E-03	3.83E-03
	0.483	4.13E-03	3.80E-03
	0.529	2.56E-03	2.61E-03
	0.126	8.10E-04	5.95E-04
	0.106	6.73E-04	6.02E-04
	0.035	5.94E-04	3.97E-04

Biology Open • Supplementary information

	0.063	3.25E-04	3.31E-04
	0.108	8.98E-04	6.23E-04
	0.063	3.25E-04	3.31E-04
	0.108	8.98E-04	6.23E-04
	0.043	6.08E-04	3.88E-04
Towering	0.630	2.87E-03	2.47E-03
	0.810	4.18E-03	3.79E-03
	0.460	2.49E-03	2.55E-03
	1.010	4.95E-03	3.88E-03
	1.200	6.15E-03	5.89E-03
	2.030	1.26E-02	1.00E-02
	0.037	7.28E-04	4.52E-04
Rafting	0.903	6.81E-03	7.05E-03
	0.510	4.80E-03	4.18E-03
	0.685	4.70E-03	5.66E-03
	0.429	2.35E-03	2.56E-03
	0.344	3.62E-03	3.13E-03
	0.725	4.34E-03	5.53E-03
	0.851	5.36E-03	5.56E-03
	0.278	2.31E-03	2.02E-03
	0.157	1.53E-03	1.13E-03
	0.046	5.71E-04	3.43E-04
	0.066	4.26E-04	4.24E-04
	0.093	1.14E-03	9.75E-04
	0.055	8.17E-04	6.58E-04
	0.141	9.65E-04	8.87E-04
	0.500	4.25E-03	3.30E-03
	0.452	2.28E-03	2.29E-03
	0.580	3.02E-03	2.69E-03

Biology Open • Supplementary information
